# Benzo[*a*]pyrene Reduces Testosterone Production in Rat Leydig Cells via a Direct Disturbance of Testicular Steroidogenic Machinery

**DOI:** 10.1289/ehp.1003391

**Published:** 2011-07-07

**Authors:** Jin-Yong Chung, Yoon-Jae Kim, Ji Young Kim, Seung Gee Lee, Ji-Eun Park, Won Rok Kim, Yong-Dal Yoon, Ki Soo Yoo, Young Hyun Yoo, Jong-Min Kim

**Affiliations:** 1 Department of Anatomy and Cell Biology, College of Medicine,; 2 Medical Science Research Center, and; 3 Mitochondria Hub Regulation Center, Dong-A University, Busan, Korea; 4 Department of Life Science, College of Natural Sciences, Hanyang University, Seoul, Korea

**Keywords:** benzo[*a*]pyrene, endocrine disruptor, epididymal sperm, Leydig cells, steroidogenesis, testosterone

## Abstract

Background: Benzo[*a*]pyrene (B[*a*]P), a polycyclic aromatic hydrocarbon (PAH), is a ubiquitous environmental pollutant that is currently suspected of being an endocrine disruptor. The testis is an important target for PAHs, yet insufficient attention has been paid to their effects on steroidogenesis in Leydig cells.

Objective: We hypothesized that long-term exposure to low concentrations of B[*a*]P might disrupt testosterone production in Leydig cells via an alteration of steroidogenic proteins.

Results: Oral exposure to B[*a*]P reduced serum and intratesticular fluid testosterone levels in rats. However, we did not observe serious testicular atrophy or azoospermia, although spermatogonial apoptosis was significantly increased. Compared with control cells, Leydig cells primed with B[*a*]P *in vivo* produced less testosterone in response to human chorionic gonadotropin (hCG) or dibutyl cyclic adenosine monophosphate *in vitro*. Of note, the reduction of testosterone levels was accompanied by decreased expression of steroidogenic acute regulatory protein (StAR) and 3β-hydroxysteroid dehydrogenase (3β-HSD), as well as increased levels of cytochrome P450 side chain cleavage (P450scc), in Leydig cells. The up-regulation of P450scc expression after exposure to B[*a*]P appears to be associated with a compensatory mechanism for producing the maximum amount of pregnenolone with the minimum amount of transported cholesterol by StAR; the down-regulation of 3β-HSD may occur because B[*a*]P can negatively target 3β-HSD, which is required for testosterone production.

Conclusions: B[*a*]P exposure can decrease epididymal sperm quality, possibly by disturbing testosterone levels, and StAR may be a major steroidogenic protein that is targeted by B[*a*]P or other PAHs.

Polycyclic aromatic hydrocarbons (PAHs) are widespread environmental and occupational contaminants formed by the incomplete combustion of organic material (International Agency for Research on Cancer 1983). Epidemiological studies carried out in occupational settings have shown that exposure to these chemicals may lead to serious diseases such as lung cancer. Among the carcinogenic PAHs, benzo[*a*]pyrene (B[*a*]P) is the best studied, and in several studies it has served as a model for the carcinogenic and mutagenic effects of PAHs. B[*a*]P is found in significant amounts in diesel exhaust, cigarette smoke, charcoal-broiled foods, and industrial waste by-products (Hattemer-Frey and Travis 1991). Although B[*a*]P released into the environment can accumulate in human tissues to toxic levels within a short time (Agency for Toxic Substances and Disease Registry 1995), the causal relationship between exposure to this agent and potential reproductive problems in humans remains uncertain.

Previous studies have shown that the testis is an important target organ for various drugs and chemicals (Blazak et al. 1985; Mattison et al. 1990). However, little attention has been given to the effect that drugs and chemicals have on steroidogenesis in Leydig cells, even though this may directly affect sperm production and quality. The effect of subacute exposure to inhaled B[*a*]P on changes in testosterone and luteinizing hormone (LH) levels and epididymal sperm has been evaluated in Fisher 344 rats (Archibong et al. 2008; Inyang et al. 2003). However, the precise cellular and biochemical mechanism(s) by which B[*a*]P affects steroidogenesis in Leydig cells has not yet been identified.

Leydig cells, testosterone-producing cells located in the interstitial compartment of mammalian testis, support spermatogenesis in seminiferous tubules (Saez 1994). In the rat testis, LH binds to Leydig cell receptors and initiates the activation of adenylate cyclase, resulting in an attendant increase in cAMP production. Steroidogenic acute regulatory protein (StAR) transfers cholesterol from the outer membrane to the inner mitochondrial membrane (Stocco 2001), where the enzyme cytochrome P450 side chain cleavage (P450scc) resides. P450scc converts cholesterol into pregnenolone, which is ultimately transferred to smooth endoplasmic reticulum, where the synthesis of testosterone takes place via the actions of 3β-hydroxysteroid dehydrogenase (3β-HSD), 17α-hydroxy/C_17–20_lase (P450c17; CYP17A1), and 17β-hydroxysteroid dehydrogenase (17β-HSD).

We hypothesized that long-term exposure to low concentrations of B[*a*]P might disrupt testosterone production by Leydig cells via the alteration of steroidogenic proteins or enzymes, such as StAR, P450scc, and 3β-HSD. To test this hypothesis, we examined the expression of these proteins in relationship to testosterone production in Leydig cells and evaluated spermatogenesis and alteration of sperm occurring in the testis and epididymis.

## Materials and Methods

*Materials.* We purchased anti-actin, anti-LH, anti–β-tubulin, and B[*a*]P from Sigma (St. Louis, MO, USA); collagenase type III from Worthington Biochemical Corp. (Lakewood, NJ, USA); Moloney murine leukemia virus (MMLV) reverse transcriptase and Taq DNA polymerase from Promega (Madison, WI, USA); and anti-P450scc and anti-ADAM3 (disintegrin and metallopeptidase domain 3) antibodies from Chemicon (Temecula, CA, USA). Anti-StAR antibody was purchased from Abcam (Cambridge, UK), and anti–3β-HSD and anti-CYP17A1 antibodies were obtained from Santa Cruz Biotechnology (Santa Cruz, CA, USA).

*Animals and B[*a*]P treatment.* Adult male Sprague-Dawley rats [8 weeks of age; 250–300 g body weight (BW)] were housed in a climate-controlled (21 ± 2°C) animal room at a constant 12/12-hr light/dark cycle, with free access to rat chow. All procedures were performed in accordance with protocols approved by the Dong-A University Animal Care and Use Committee. Animals were treated humanely and with regard for alleviation of suffering. The rats received B[*a*]P for 90 days by daily gavage at doses of 0.001, 0.01, or 0.1 mg/kg BW per day (*n* = 20–25 rats per dose). Control animals (*n* = 25) received the same weight-based volume of vehicle (0.5% DMSO). On day 90, the rats were sacrificed and the testes and epididymides were removed. The left testis was used to collect interstitial fluid (IF), and the right testis was used for subsequent Leydig cell isolation. For histological studies, the rats were anesthetized with sodium pentobarbital, and testes were fixed by perfusing Bouin’s fixative through the dorsal aorta.

*Collection of IF and hormone assays.* IF was collected according to a previously described method (Turner et al. 1984). We determined testosterone concentrations in serum, IF, and culture medium for duplicate samples using a testosterone ELISA kit (IBL, Hamburg, Germany) following the manufacturer’s instructions. The sensitivity of the assay was 0.083 ng/mL, and the intra- and interassay coefficients of variation (CVs) were 3.3% and 6.7%, respectively. LH concentration in serum was determined for duplicate samples using an LH ELISA kit (LH Detect®; INRA, Nouzilly, France). The sensitivity of the assay was 0.01 ng/mL, and the intra- and interassay CVs were 4% and 8%, respectively.

*Leydig cell isolation and culture.* Leydig cells were isolated as described previously (Klinefelter et al. 1987), with slight modifications. The testes were decapsulated, placed in a dissociation buffer [M-199 medium with 2.2 g/L HEPES, 1.0 g/L bovine serum albumin (BSA), 2.2 g/L sodium bicarbonate (pH 7.4), and 25 mg/L trypsin inhibitor] containing collagenase (0.25 mg/mL) at 34°C, and shaken for 30 min. Digested testes were passed through a 100-μm nylon mesh, and Leydig cells were purified by Percoll gradient separation. The final purity of the Leydig cells, determined by staining the cells for 3β-HSD activity, was consistently approximately 90%.

We performed the Leydig cell culture as described previously (Klinefelter and Ewing 1988), with minor modifications. Briefly, isolated Leydig cells were resuspended in M-199 medium containing 15 mM HEPES, 0.1% BSA, 5 μg/mL gentamicin, 50 U/mL penicillin, and 50 μg/mL streptomycin. The cells (1.0 × 10^6^) were then added to a six-well culture plate and cultured for 24 hr without serum in the absence or presence of hCG (25 IU/mL) or dibutyl cyclic adenosine monophosphate (dbcAMP; 50 μM) at 34°C in 5% CO_2_:95% air.

*Immunocytochemistry and immunohistochemistry.* Isolated Leydig cells were attached to the glass slide by cytospin centrifugation. The cells were fixed with 4% paraformaldehyde, washed with phosphate-buffered saline (PBS), and incubated with 0.2% Triton X-100. Then, the cells were incubated with the appropriate primary antibody in 1% BSA at room temperature (RT). For secondary antibody reactions, the cells were incubated with an appropriate fluorescence-conjugated secondary antibody at RT. Finally, cells were mounted and observed under a confocal microscope (LSM510; Carl Zeiss, Hamburg, Germany).

For immunohistochemistry, deparaffinized and hydrated testis and pituitary gland sections were treated with 3% hydrogen peroxide (H_2_O_2_) for 5 min, rinsed with PBS for 15 min, and then immunostained using the Vectastain ABC kit (Vector Laboratories, Burlingame, CA, USA) following the manufacturer’s instructions.

*Terminal deoxy-nucleotidyl transferase-mediated digoxigenin-deoxyuridine triphosphate nick end labeling (TUNEL).* Sections of testis were deparaffinized, hydrated, treated in 3% H_2_O_2_ for 5 min, and rinsed with PBS for 15 min. We then quantified apoptosis using the In Situ Cell Death Detection Kit, POD (peroxidase; Roche, Penzberg, Germany) following the manufacturer’s instructions. TUNEL-positive germ cells in the seminiferous tubules were counted, ensuring that there were at least 20 tubules per middle portion of the testis section in each group.

*Histological staining of the epididymis.* Paraffin sections of the epididymis were deparaffinized, stained with periodic acid Schiff’s (PAS) reagent (Sigma-Aldrich, St. Louis, MO, USA), counterstained with hematoxylin, and observed with a ScanScope digital slide scanning system (Aperio, Vista, CA, USA). We measured diameters of the caput and cauda epididymal tubules (35 tubules/section from five samples of each group) using ImageScope viewer software (Version 9.1.19.1569; Aperio).

*Sperm preparation and analysis.* Sperm obtained from the portion of the cauda epididymis that was stained were released into an equal amount of Dulbecco’s PBS (DPBS). For sperm number and motility count, 200 μL of sperm sample in DPBS was applied to a hemocytometer. Sperm counts were expressed as the sum of five squares on the hemocytometer, and the percent motility was determined by counting both motile and immotile spermatozoa per area. For sperm acrosomal integrity analysis, sperm were stained with LysoTracker DND-26 (Invitrogen, Carlsbad, CA, USA) at RT for 30 min. For ADAM3 immunocytochemistry, sperm were attached to a glass slide by cytospin centrifugation, and the attached sperm samples were fixed with methanol at RT for 5 min and immunostained with anti-ADAM3 antibody. Sperm nuclei and mitochondria were counterstained with Hoechst 33258 and MitoTracker (Invitrogen), respectively.

*Western blot analysis.* We prepared Leydig cell lysates by incubating cell pellets in lysis buffer [30 mM NaCl, 0.5% Triton X-100, 50 mM Tris-HCl (pH 7.4), 1 mM Na_3_VO_4_, 25 mM NaF, 10 mM Na_4_P_2_O_7_] for 30 min on ice. After the insoluble fractions were removed, the supernatants were collected, and protein concentration was determined with a bicinchoninic acid (BCA) protein assay kit (Pierce Biotechnology, Woburn, MA, USA). Aliquots of protein (~ 30 μg) were subjected to SDS-PAGE and transferred onto nitrocellulose membranes. The membranes were incubated for 1 hr at RT with a primary antibody in Tris-buffered saline containing 0.05% Tween-20 (TBS-T; pH 7.4) in the presence of 5% nonfat dry milk. After the membranes were washed in TBS-T, they were incubated with horseradish peroxidase–conjugated secondary antibody. The signals were detected with an enhanced chemiluminescence detection kit (Amersham Pharmacia Biotech, Piscataway, NJ, USA) in the LAS-3000 detector (Fujifilm, Tokyo, Japan).

*Reverse-transcription polymerase chain reaction (RT-PCR).* Total cellular RNA was isolated from isolated Leydig cells using Trizol reagent (Invitrogen). The cDNA was synthesized from 5 μg total RNA using an oligo-dT random primer and MMLV RNase H^–^ reverse transcriptase. cDNA (3 μL) was subjected to PCR (18 cycles) for P450scc, StAR, and 3β-HSD in 30 μL reaction mixture [10× PCR buffer, 2.5 mM dNTP (deoxynucleotide triphosphate), Taq-polymerase 5 U, and upstream and downstream primers; see Supplemental Material, Table 1 (http://dx.doi.org/10.1289/ehp.1003391)]. The PCR products were resolved by 2% agarose gel electrophoresis and visualized using ethidium bromide.

*Statistical analysis.* Data are expressed as the mean ± SD of three or four separate experiments. Where appropriate, data were subjected analysis of variance followed by Duncan’s post hoc test. Means were considered significantly different at *p* < 0.05.

## Results

**Figure 1:**
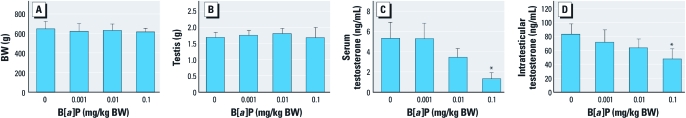
Effect of B[*a*]P treatment on BW (*A*), testis weight (*B*), and testosterone concentrations in serum (*C*) and intratesticular fluids (*D*). Testosterone levels were measured by ELISA (*C* and *D*). Data are mean ± SD (*n* = 9). *p < 0.05 compared with control.

*Effect of B[*a*]P on body and testicular weights and testosterone concentrations in serum and intratesticular fluids.* To investigate whether exposure to B[*a*]P provoked the disruption of the male reproductive endocrine system, we treated adult Sprague-Dawley rats with B[*a*]P (0.001, 0.01, or 0.1 mg/kg BW/day) for 90 days by gavage. Compared with DMSO treatment, none of the B[*a*]P doses significantly altered BW (Figure 1A) or testicular weight (Figure 1B). However, testosterone concentrations in both serum and intratesticular were reduced after treatment with B[*a*]P, most remarkably in the 0.1 mg/kg group (Figure 1C,D).

**Figure 2:**
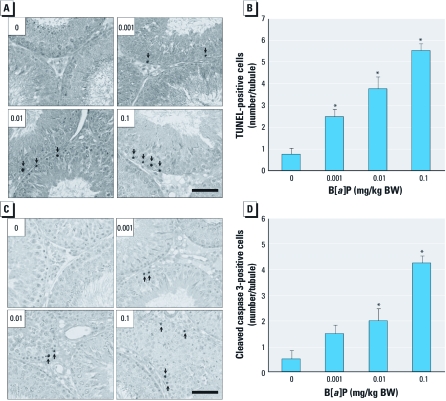
Incidence of apoptotic germ cell death after treatment with B[*a*]P (0, 0.001, 0.01, or 0.1 mg/kg BW/day) for 90 days. (*A*) TUNEL staining of testes (original magnification, 100×) and (*B*) quantitation of TUNEL-positive germ cells in seminiferous tubules. (*C*) Immunolocalization of active caspase-3 in testes (original magnification: 400×) and (*D*) quantitation of active caspase-3–positive germ cells in seminiferous tubules. In *A* and *C*, arrows indicate TUNEL-positive germ cells and active caspase-3-positive germ cells, respectively; bar = 100 µm. In *B* and *D*, values shown are mean ± SD (*n* = 180 tubules from nine testes). *p < 0.05 compared with control.

**Figure 3:**
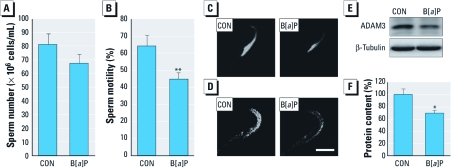
Alterations in sperm number, sperm motility, and qualitative markers of sperm after treatment with B[*a*]P (0, 0.001, 0.01, or 0.1 mg/kg BW/day) for 90 days. CON and B[*a*]P indicate 0 mg/kg BW (control) and 0.01 mg/kg BW groups, respectively. Change (mean ± SD) in sperm number (*A*) and sperm motility (*B*). (*C*) Changes in acrosomal integrity evaluated by LysoTracker DND-26 fluorescence staining (original magnification, 1,600×). (*D* and *E*) Changes in ADAM3 proteins detected by immunocytochemistry (*D*; original magnification, 1,600×; bar = 5 μm) and (*E*) Western blotting. (*F*) Densitometric quantification of ADAM3 protein content in sperm protein extracts (mean ± SD). *p < 0.05, and **p < 0.01 compared with control.

*Occurrence of germ cell apoptosis and sperm changes in the epididymis after B[*a*]P treatment.* The withdrawal of testosterone, especially intratesticular testosterone, causes germ cell apoptosis and thereby depletion of spermatozoa in the testis (Kim et al. 2001). Thus, we analyzed the occurrences of testicular germ cell apoptosis and sperm changes in the epididymis. In the B[*a*]P-treated groups, the number of apoptotic germ cells detected by TUNEL increased (Figure 2A) in a dose-dependent manner (Figure 2B); in contrast, TUNEL-positive cells were rare in controls (Figure 2A). Consistent with these results, activated caspase-3 proteins were positively stained in the apoptotic germ cells of testes in the B[*a*]P-exposed groups but not in controls (Figure 2C,D). We did not detect either TUNEL-positive or active caspase-3–positive staining in other testicular cell types (i.e., Leydig cells or Sertoli cells) in any of the groups. The apoptotic germ cells formed after exposure to B[*a*]P were mostly restricted to the spermatogonia; other types of germ cells in the spermatogenic lineage appeared to be intact. In the epididymis, exposure to B[*a*]P resulted in a noticeable reduction in diameter of caput epididymal tubules [see Supplemental Material, Figure 1A,B (http://dx.doi.org/10.1289/ehp.1003391)] as well as that of the cauda epididymal tubules (see Supplemental Material, Figure 1C,D). Epididymal sperm numbers appeared to be lower in B[*a*]P-treated groups than in controls, but the difference was insignificant (Figure 3A). However, sperm motility was significantly reduced in the B[*a*]P-exposed groups compared with controls (Figure 3B). Qualitatively, acrosomal integrity detected by LysoTracker DND-26 staining was remarkably reduced in sperm heads from the cauda epididymis of rats exposed to B[*a*]P compared with controls (Figure 3C; see also Supplemental Material, Figure 2A). Furthermore, protein analysis of ADAM3, a sperm surface protein associated with the fertilization process (Nishimura et al. 2004), revealed that this protein was down-regulated in the sperm surfaces of B[*a*]P-treated groups (Figure 3D; see also Supplemental Material, Figure 2B), and its content was significantly decreased in B[*a*]P-exposed groups compared with controls (Figure 3E,F).

**Figure 4:**
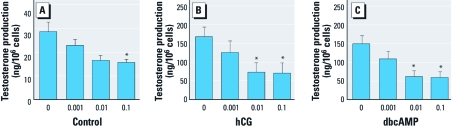
Testosterone production capability of testicular Leydig cells from rats exposed to B[*a*]P. Leydig cells were isolated from testes of B[*a*]P-exposed rats and cultured for 24 hr in the absence (control; *A*) or presence of hCG (25 IU/mL; *B*) or dbcAMP (50 μM; *C*). Testosterone levels in media (mean ± SD) were measured by ELISA and represent three independent experiments performed in triplicate. *p < 0.05 compared with control.

*Capability for testosterone production is reduced in Leydig cells that are exposed* in vivo *to B[*a*]P.* To monitor their testosterone production capability, we cultured Leydig cells isolated from the testes of controls and B[*a*]P-exposed rats in the absence or presence of hCG or dbcAMP. hCG or dbcAMP remarkably stimulated testosterone production in control Leydig cells (Figure 4). However, hCG- or dbcAMP-stimulated testosterone production was lower in cells primed with B[*a*]P *in vivo*; this was most evident in the groups treated with 0.01 and 0.1 mg/kg BW (Figure 4B,C). In addition, spontaneous testosterone production in cultures without hCG or dbcAMP was also reduced in the B[*a*]P-primed cells, although the differences with respect to controls were relatively smaller than those in hCG or dbcAMP-treated cultures (Figure 4A).

**Figure 5:**
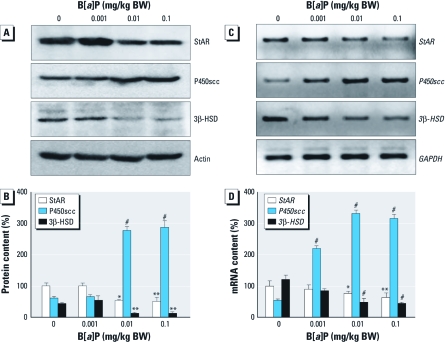
Changes in StAR, P450scc, and 3β-HSD protein and gene expression in testicular Leydig cells from rats exposed to B[*a*]P. GAPDH, glyceraldehyde 3-phosphate dehydrogenase. (*A*) Western blot analyses for StAR, P450scc, and 3β-HSD and (*B*) densitometric quantification (mean ± SD) of these proteins in Leydig cell protein extracts. (*C*) RT-PCR analyses for gene expression of *StAR*, *P450scc*, and *3*β*-HSD* and (*D*) densitometric quatification (mean ± SD) of *StAR*, *P450scc*, and *3*β*-HSD* mRNA contents in Leydig cell RNA extracts. *p < 0.05, **p < 0.01, and ^#^*p* < 0.001, compared with the corresponding control.

**Figure 6:**
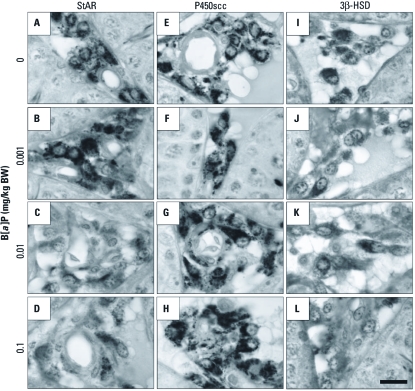
Immunohistochemical localization of StAR (*A–D*), P450scc (*E–H*), and 3β-HSD (*I–L*) in the testicular interstitial compartments of rats treated with B[*a*]P at 0 (*A,E,I*), 0.001 (*B,F,J*), 0.01 (*C,G,K*), or 0.1 (*D,H,L*) mg/kg BW. Original magnification, 1,000×; bar = 10 μm.

*Changes in StAR, P450scc, and 3*β*-HSD expression in Leydig cells after* in vivo *exposure to B[*a*]P.* It has long been known that StAR, P450scc, and 3β-HSD are prerequisites for testosterone production in Leydig cells of the mammalian testis (Zirkin and Chen 2000). Therefore, delineating the changes of these proteins in Leydig cells is important in discovering the precise mechanism(s) by which B[*a*]P affects testosterone production. Figures 5 and 6 show the effects of B[*a*]P exposure on the expression of steroidogenic proteins in Leydig cells. Long-term exposure to B[*a*]P resulted in down-regulation of StAR and 3β-HSD expression, as shown by Western blotting (Figure 5A), RT-PCR (Figure 5C), and immunocytochemistry using isolated Leydig cells [see Supplemental Material, Figure 3 (http://dx.doi.org/10.1289/ehp.1003391)]. However, P450scc expression was substantially up-regulated after exposure to B[*a*]P [Figure 5A,C; see also Supplemental Material, Figure 3). We further confirmed these observations by immunohistochemistry of the testicular sections (Figure 6; see also Supplemental Material, Figure 4). A decrease of StAR (Figure 6C,D) and 3β-HSD (Figure 6J–L), in the Leydig cells was evident in the B[*a*]P-exposed groups. In contrast, immunoreactivity for P450scc proteins was more intense in the B[*a*]P-treated groups (Figure 6G,H) than in controls (Figure 6E). Additionally, we examined the change in CYP17A1 expression in Leydig cells, because this enzyme is particularly decreased after exposure to exobiotic toxicants (Akingbemi et al. 2004). Exposure to B[*a*]P caused a significant decrease in CYP17A1 expression in Leydig cells at both translational and transcriptional levels (see Supplemental Material, Figure 4).

**Figure 7:**
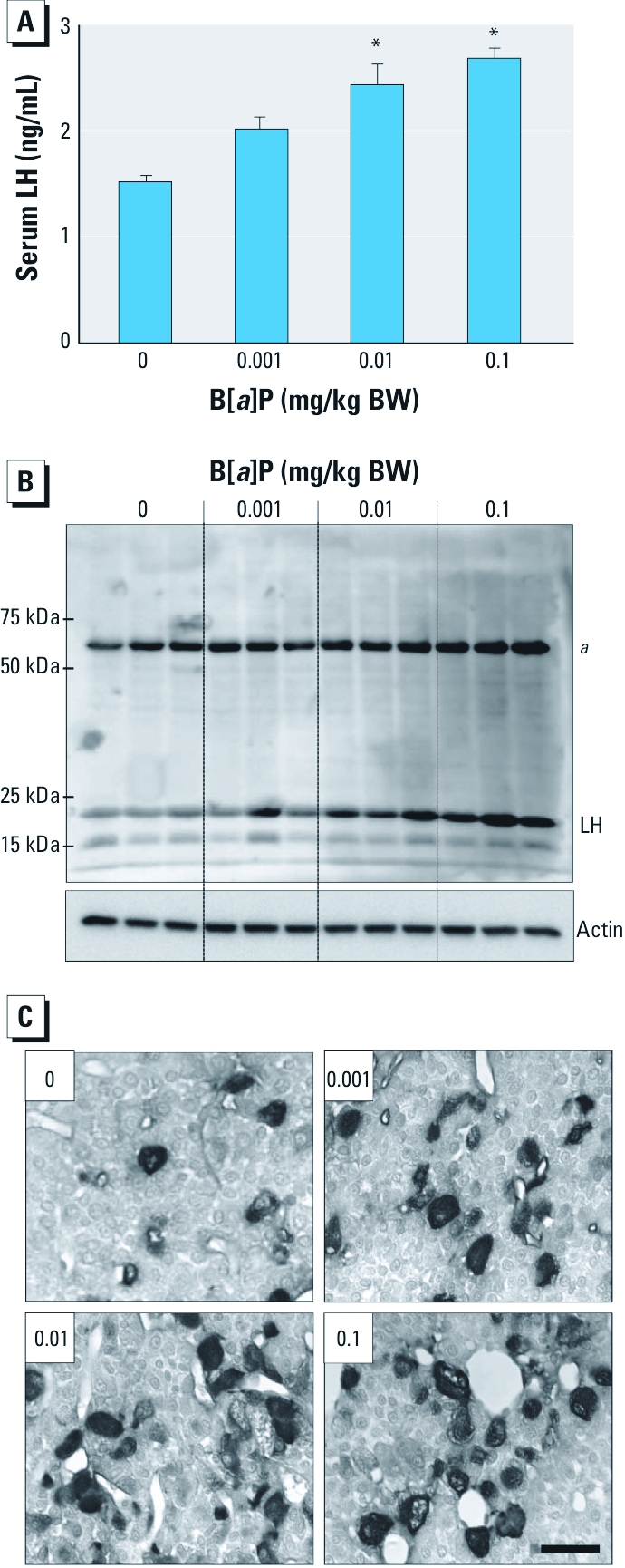
Effects of exposure to B[*a*]P (0, 0.001, 0.01, and 0.1 mg/kg/BW) on LH serum levels and protein expression in the pituitary glands of B[*a*]P-exposed rats. (*A*) LH levels measured in serum by ELISA (mean ± SD; *n* = 9). (*B*) Western blot analysis of protein extracts from pituitary glands. (*C*) Immunohistochemical localization of LH in the anterior pituitary glands. Original magnification: 400×; bar = 20 μm. ***^a^***Nonspecific band. *p < 0.05 compared with the control.

*Chronic exposure to B[*a*]P does not directly affect LH release from the pituitary gland.* We initiated this study on the basic premise that chronic exposure to B[*a*]P results in a decrease in testosterone production due to disturbances in the steroidogenic machinery of Leydig cells. However, it is still uncertain whether the testosterone reduction is due to decreased LH levels arising from the direct and negative effects of B[*a*]P on the neuroendocrine system. To assess this possibility, we measured LH concentrations in serum and monitored expression levels of LH protein in tissue samples from the pituitary glands. Exposure to B[*a*]P resulted in significant increases in serum LH levels (Figure 7A) and LH proteins (~ 20 kDa) in pituitary gland extracts (Figure 7B) and in the intensity of LH immunoreactivity in the gonadotropes in the anterior pituitary glands (Figure 7C).

## Discussion

B[*a*]P is known to have xenoestrogenic action (Santodonato 1997) and is currently considered to be an endocrine disruptor (Hoyer 2001). B[*a*]P is present in cigarette smoke at a concentration of 20–40 ng/cigarette (Kuller et al. 1986); coke oven workers are exposed to approximately 42 μg B[*a*]P/m^3^ (Lewtas et al. 1997); and indoor exposure to B[*a*]P from cooking oil fumes reaches 20 μg B[*a*]P/m^3^ (Chiang et al. 1997). Nevertheless, the effect of chronic exposure to B[*a*]P on hormonal dysregulation in various mammalian endocrine glands has not yet been investigated. Furthermore, the precise cellular and biochemical mechanism(s) by which B[*a*]P disrupts the reproductive endocrine system should be determined. The exposure levels of B[*a*]P used in our study (0.5–50 ng/day for an average rat weighing 0.5 kg) are considered environmentally relevant, because B[*a*]P intake has been estimated to range from 20 to 800 ng/day in humans (0.3–13.3 ng/day for an average man weighing 60 kg) who lives near hazardous waste sites contaminated with PAHs (Lioy et al. 1988). In the present study, we found that oral exposure to low concentrations of B[*a*]P reduced testosterone production in Leydig cells via alterations in the levels of steroidogenic proteins (especially down-regulation of StAR), which led to reduced sperm quality in the epididymis.

In mammalian male reproduction, testosterone, produced by Leydig cells in response to LH, plays a pivotal role in the initiation and maintenance of spermatogenesis by affecting Sertoli cell androgen receptors. Thus, an abnormal reduction in serum or intratesticular fluid testosterone levels can cause testicular atrophy, which is accompanied by a decrease in the number of germ cells and, ultimately, azoospermia (Awoniyi et al. 1989). In the present study, oral exposure to B[*a*]P resulted in reduction of serum and intratesticular fluid testosterone levels. However, although both serum and intratesticular testosterone levels were decreased, testicular atrophy or azoospermia did not occur; this indicates that the reduction in testosterone levels was not sufficient to induce massive germ cell degeneration. In fact, it has been shown that a lower concentration (< 20 ng/mL) of intratesticular testosterone needs to be present for at least 4 weeks to achieve azoospermia in rat testes (Kim et al. 2001; McLachlan et al. 1994). However, in the present study, the intratesticular testosterone level in the group treated with the highest dose of B[*a*]P (0.1 mg/kg BW) dropped to around 50 ng/mL. Based on the extent of apoptosis we observed, we cannot exclude the possibility that germ cell (mostly spermatogonia) apoptosis might be provoked by the direct cytotoxic effect of B[*a*]P treatment. Indeed, exposure to many types of chemical agents is known to induce spermatogonial apoptosis with no change in testosterone concentration (Boekelheide and Hall 1991; Meistrich et al. 2003).

Although we observed no serious spermatogenic effects after B[*a*]P exposure in the present study, the reduced testosterone levels appeared to be more closely correlated with decreased sperm quality than with the occurrence of germ cell apoptosis. In the epididymis, testosterone is a critical substance because it is converted to dihydrotestosterone by 5α-reductase (Robaire et al. 1977). Therefore, testosterone is very important for the regulation of epididymal epithelial cell function as well as for maturation of spermatozoa. Our results indicate that B[*a*]P can disrupt testosterone production in Leydig cells, which may ultimately lead to a decrease in sperm quality in the epididymis via reduced levels of sperm motility, acrosomal integrity, and fertility-related proteins such as ADAM3 (Nishimura et al. 2004).

The rate-limiting enzymatic reaction in steroidogenesis is catalyzed by P450scc (Kahnt et al. 1974). However, it has been suggested that the physiologically relevant rate-limiting step is the transfer of free cholesterol from the outer mitochondrial membrane to the inner membrane where the P450scc resides (Stocco and Chen 1991). Currently, this transfer is thought to be mediated in part by the StAR protein (Stocco 2001). The present study is the first to show that long-term exposure to B[*a*]P decreases *StAR* expression and increases *P450scc* expression in Leydig cells. We found that B[*a*]P-primed Leydig cells *in vivo* produced less testosterone in response to hCG or dbcAMP *in vitro* compared with control cells. Given that testosterone production decreases after exposure to B[*a*]P, it may be assumed that StAR is one of the major target proteins affected by B[*a*]P during testosterone synthesis. In fact, the decrease in *StAR* expression in isolated Leydig cells after exposure to B[*a*]P was completely restored by supplementation with hCG [see Supplemental Material, Figure 5 (http://dx.doi.org/10.1289/ehp.1003391)], and elevated testosterone production upon hCG or dbcAMP stimulation remained static at all concentrations of B[*a*]P. These results support evidence that StAR may be one of the major steroidogenic proteins involved in the disturbance of testosterone production in Leydig cells after exposure to B[*a*]P.

In the present study, P450scc expression in Leydig cells was up-regulated after exposure to B[*a*]P (Figures 5, 6); this appears to be associated with a compensatory phenomenon related to producing the maximal amount of pregnenolone with a minimal amount of transported cholesterol by the reduced StAR content, but the association remains uncertain at present. We also found that 3β-HSD expression was decreased in Leydig cells after exposure to B[*a*]P. The HSDs are essential for steroidogenesis in Leydig cells (Payne and Youngblood 1995). 3β-HSD, 17α-hydroxylase, and 17β-HSD are required for the synthesis of steroids. 11β-HSD, 3α-HSD, and 5α-reductase are also involved in the synthesis, as well as metabolism, of steroids. Among these, 3β-HSD is considered to be a key enzyme necessary for the synthesis of testosterone in Leydig cells of rats (Haider et al. 1986). In the present study, the down-regulation of 3β-HSD expression after B[*a*]P exposure did not appear to be the result of an arbitrary cytotoxic action of B[*a*]P because P450scc expression was up-regulated by B[*a*]P. 17α-Hydroxylase (CYP17A1) in rat Leydig cells has been shown to be very sensitive to exobiotic toxicants such as bisphenol A (Akingbemi et al. 2004). Similarly, our results demonstrated that CYP17A1 expression is down-regulated in Leydig cells of rats exposed to B[*a*]P. This indicates that 3β-HSD and CYP17A1 could also be potential targets of B[*a*]P in the testicular steroidogenic machinery.

Finally, to confirm whether decreased testosterone concentration is related to the direct inhibition of neuroendocrine function by B[*a*]P, we monitored the changes in the serum LH level and expression of LH proteins in the pituitary glands. Unexpectedly, chronic B[*a*]P exposure significantly increased both serum LH concentration and LH protein expression in the pituitary glands. Similarly, an elevation in serum LH levels was recently observed in F-344 male rats after B[*a*]P inhalation (Archibong et al. 2008). Such up-regulation of LH levels is believed to be associated with the reduced negative feedback regulation triggered by decreased testosterone levels in blood circulation. Therefore, the possibility that testosterone production was reduced via direct inhibition of the neuroendocrine system after B[*a*]P exposure can be disregarded. We believe that it is not likely that the pituitary is a direct target of B[*a*]P action.

## Conclusion

Our data indicate that long-term exposure to B[*a*]P results in a significant reduction in both serum and intratesticular testosterone levels. The decrease was insufficient to cause testicular atrophy with massive germ cell apoptosis but appeared to be associated with a reduction in sperm quality in the epididymis. The down-regulation of StAR expression might be an important factor through which testosterone production is interrupted after B[*a*]P exposure. Our study suggests that B[*a*]P exposure can decrease epididymal sperm quality by reducing the testosterone level and that StAR could be an important steroidogenic protein that is targeted by B[*a*]P or other PAHs.

## Supplemental Material

(764 KB) PDF
