# Supplementary material for: Benzo[a]pyrene Reduces Testosterone Production in Rat Leydig Cells via a Direct Disturbance of Testicular Steroidogenic Machinery
Source: Environ Health Perspect. 2011 Jul 7;119(11):1569–74. doi: 10.1289/ehp.1003391 (PMC3226498; doi:10.1289/ehp.1003391)
Supplement: (764 KB) PDF [file ehp.1003391.s001.pdf]

Benzo[a]pyrene Reduces Testosterone Production in Rat Leydig Cells via a Direct Disturbance of Testicular Steroidogenic Machinery

Jin-Yong Chung, Yoon-Jae Kim, Ji Young Kim, Seung Gee Lee, Ji-Eun Park, Won Rok Kim, Yong-Dal Yoon, Ki Soo Yoo, Young Hyun Yoo, and Jong-Min Kim

Supplemental Material

Table of contents

• Supplementary Figure 1. Histological changes in the caput and cauda epididymis following exposure to B[a]P. -- 1

• Supplementary Figure 2. Acrosomal change (A) and immunolocalization of ADAM3 (B) in sperm. -- 2

• Supplementary Figure 3. Immunocytochemical localizations of StAR, P450scc, and 3β-HSD in Leydig cells.-- 3

• Supplementary Figure 4. Changes of CYP17A1 expression in Leydig cells from testes of rats exposed to B[a]P. -- 4

• Supplementary Figure 5. Recovery of decreased StAR protein content in Leydig cells exposed to B[a]P *in vivo* by hCG treatment *in vitro*. --- 5

• Supplementary Table 1. Oligonucleotide sequences used for RT-PCR --------------------------- 6

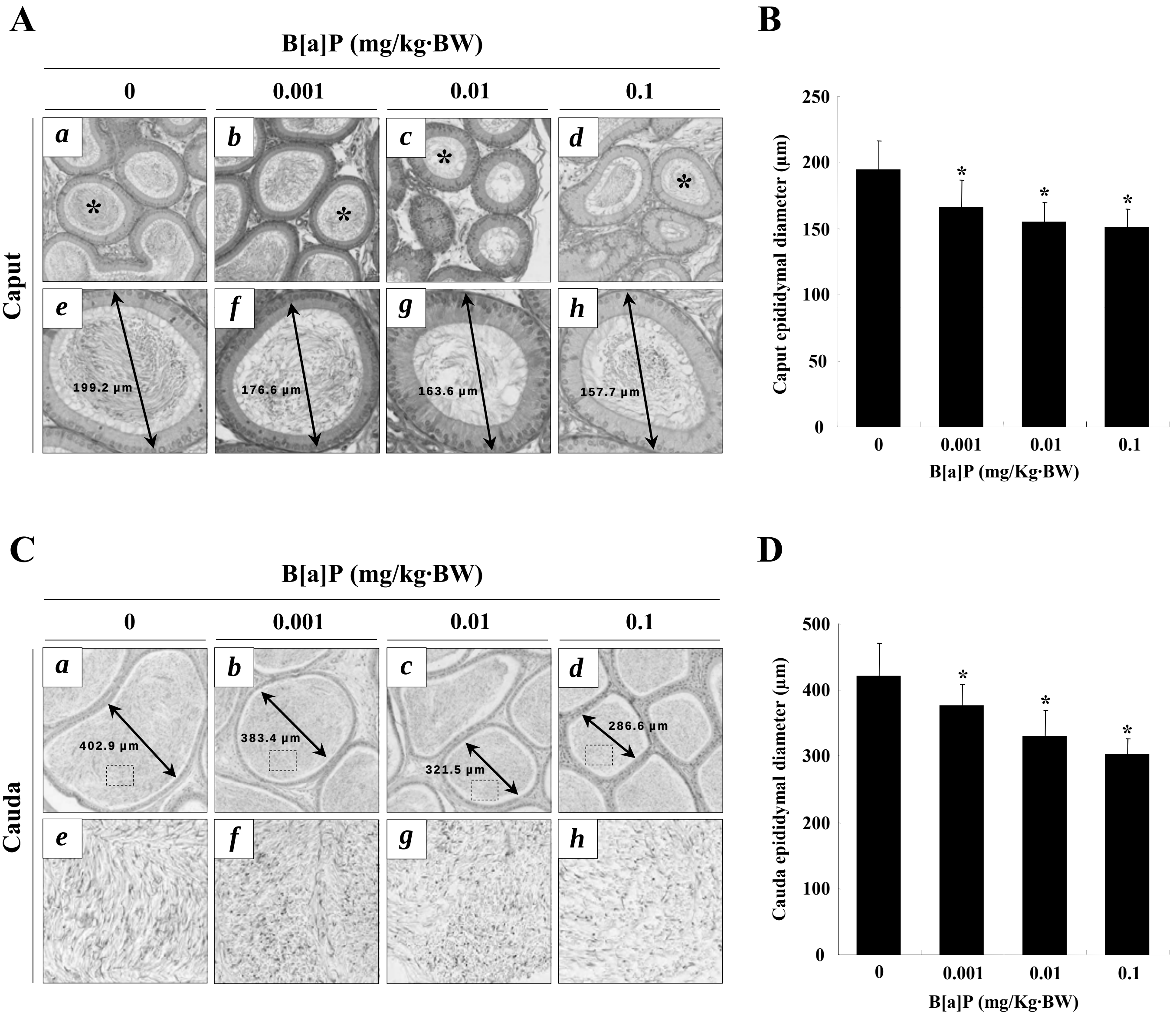

Supplementary Figure 1. Histological changes in the caput and cauda epididymis following exposure to B[a]P. Rats received 0, 0.001, 0.01, or 0.1 mg/kg-BW/day of B[a]P for 90 days by gavage. A: PAS staining of caput epididymides. B: Diagram illustrating changes in diameters (indicated by arrows in A-e, A-f, A-g, and A-h) of caput epididymal tubules. C: PAS staining of cauda epididymides. D: Diagram illustrating changes in diameters (indicated by arrows in C-a, C-b, C-c, and C-d) of caput epididymal tubules. *A and C:* a and e, control (0 mg); b and f, 0.001 mg; c and g, 0.01 mg; and d and h, 0.1 mg. Original magnification (upper panel): ×200. Photographs in the lower panel were digitally magnified 3.6× from the asterisk or dot-lined box in the upper panel. *B and D:* 175 tubules of each group (from 5 samples) were chosen for measurement of diameter. *, $P < 0.05$ compared with control.

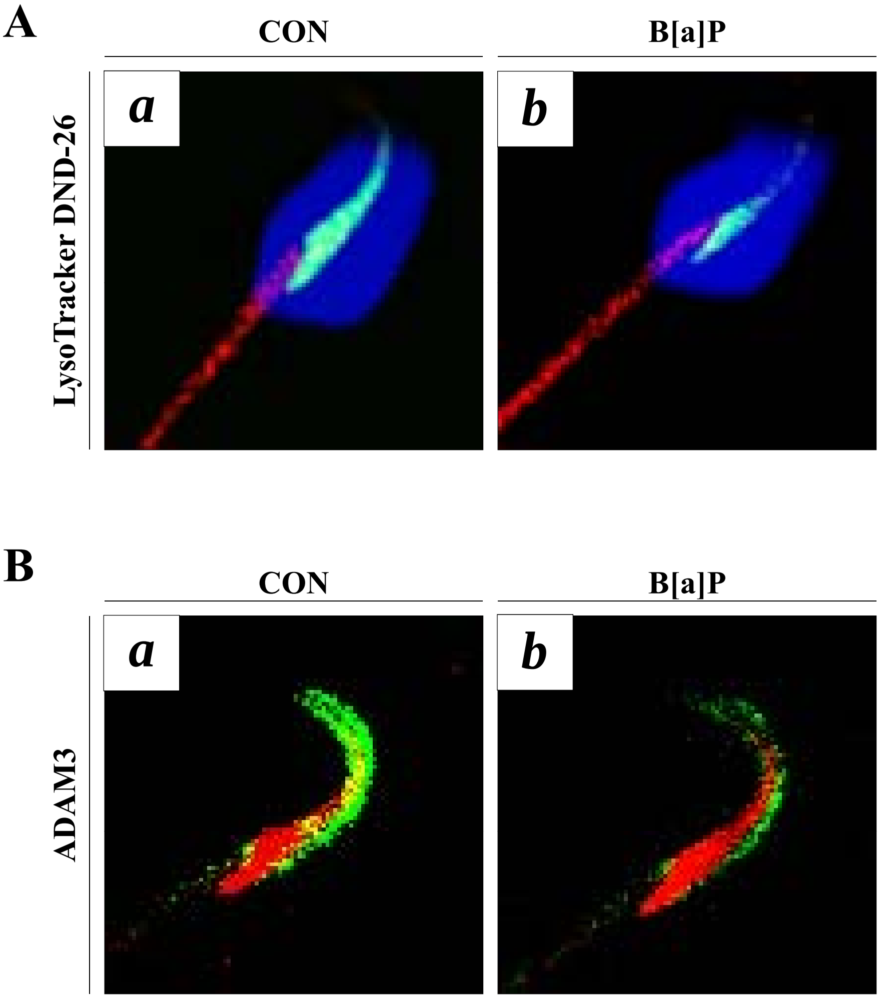

Supplementary Figure 2. Acrosomal change (A) and immunolocalization of ADAM3 (B) in sperm. A: Live sperm were stained with LysoTracker DND-26 (green) for sperm acrosomal integrity analysis. Sperm nuclei and mitochondria were counterstained with Hoechst 33258 (blue) and MitoTracker (red), respectively. Original magnification: ×1,600. B: Sperm were attached to a glass slide by cytospin centrifugation for immunocytochemistry. The attached sperm samples were fixed with methanol and immunostained with anti-ADAM3 (green) antibody. Sperm nuclei were counterstained with propidium iodide (red). Original magnification: ×1,600. The stained sperm observed using a confocal microscope.

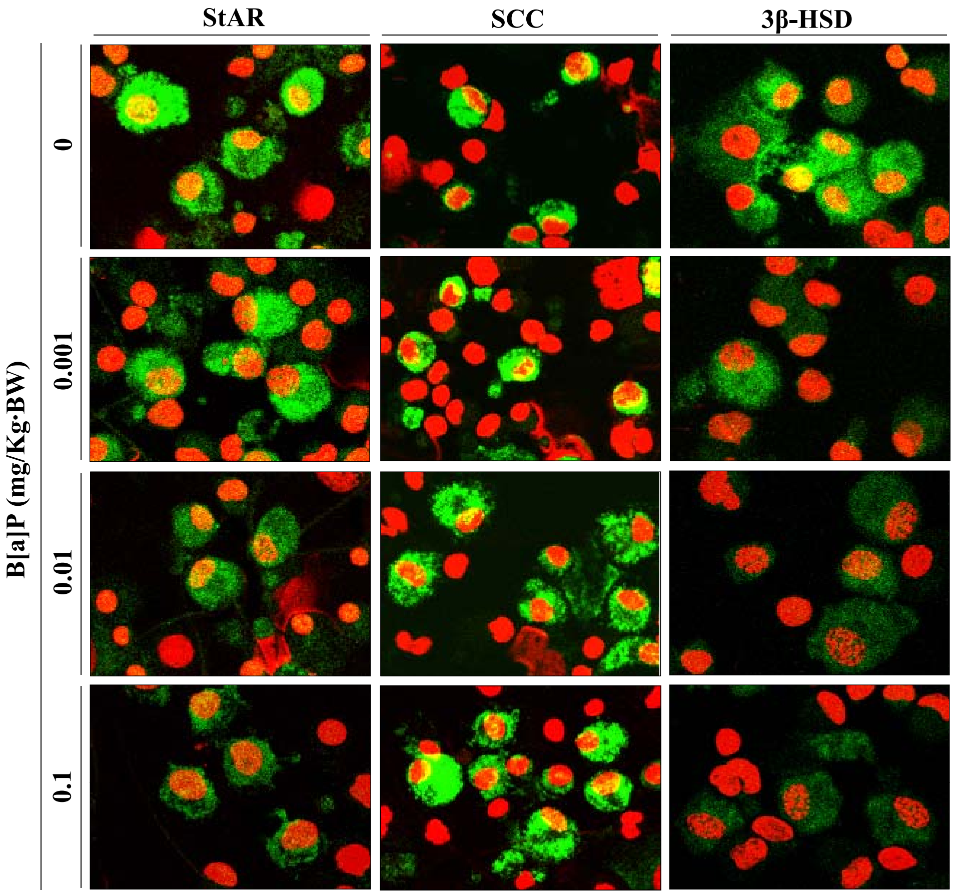

Supplementary Figure 3. Immunocytochemical localizations of StAR, P450scc, and 3β-HSD in Leydig cells. The cells were cytospun, fixed, and immunostained with the corresponding antibody, counter stained with propidium iodide, and observed using a confocal microscope. Original magnification: ×800.

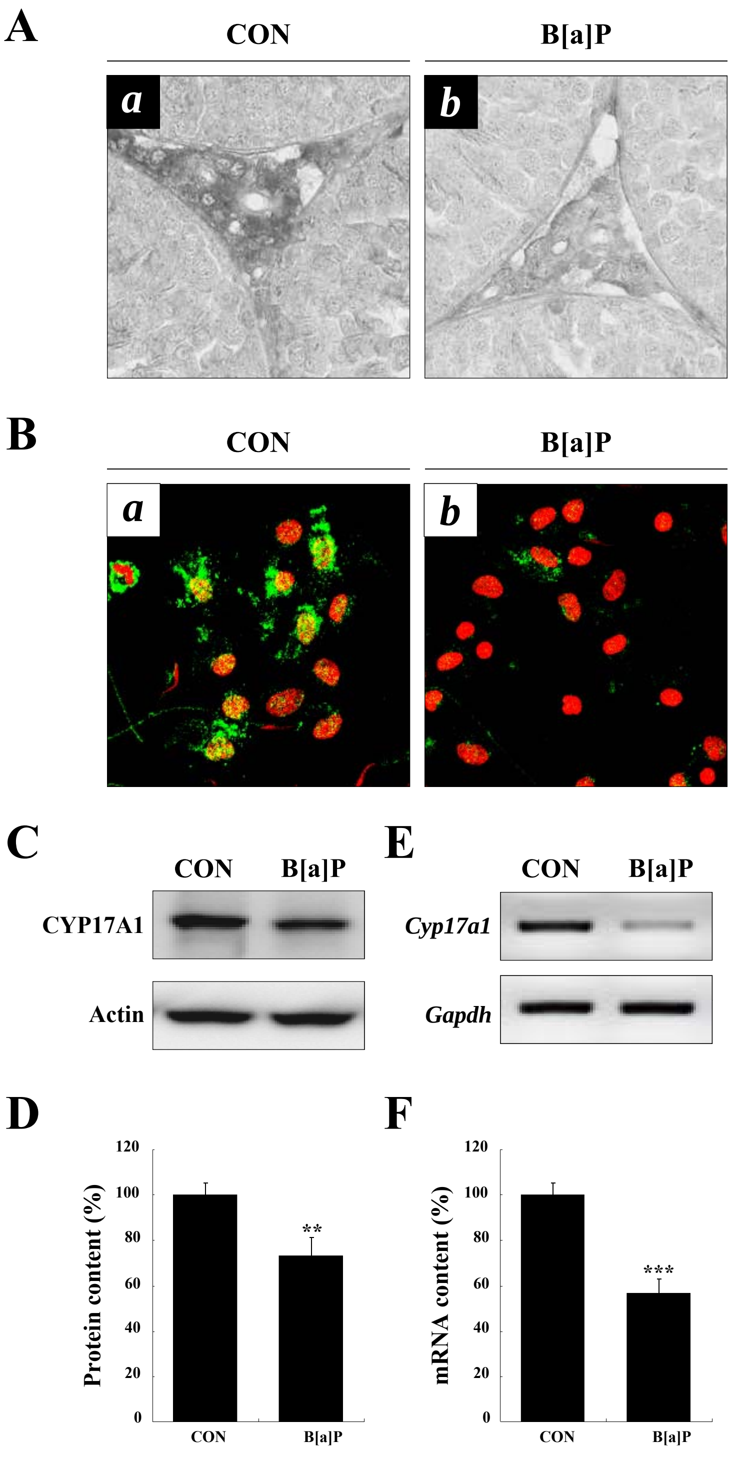

Supplementary Figure 4. Changes of CYP17A1 expression in Leydig cells from testes of rats exposed to B[a]P. Rats received 0 or 0.01 mg/Kg BW/day of B[a]P for 90 days by gavage. A: Immunohistochemical localization of CYP17A1 in testicular interstitial compartment of rats. Original magnification: ×400. B: Immunocytochemical localization of CYP17A1 in Leydig cells. Original magnification: ×200. C: Western blot analysis for CYP17A1. D: Densitometric quatification of CYP17A1protein content in Leydig cell protein extracts. E: RT-PCR analysis for CYP17A1 gene expression. Gapdh expression was examined as internal control. F: Densitometric quatification of CYP17A1 mRNA content in Leydig cell RNA extracts. In D and F, **, $P<0.01$ and ***, $P<0.001$ compared with control.

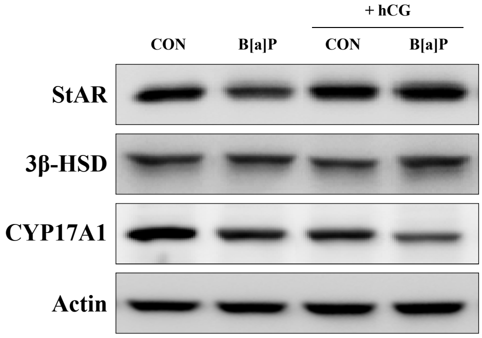

Supplementary Figure 5. Recovery of decreased StAR protein content in Leydig cells exposed to B[a]P *in vivo* by hCG treatment *in vitro*. Leydig cells were isolated from testes of rats that had received 0 and 0.01 mg/Kg BW/day of B[a]P for 90 days by gavage, and cultured for 24 h in the absence or presence of hCG (25 IU/ml). Protein extracts from harvested cells were applied to western blot analysis for StAR, 3β-HSD, and CYP17A1.

Supplementary Table 1. Oligonucleotide sequences used for RT-PCR

Gene	Primer sequences (5' -> 3')	Accession Number	References
P450scc (CYP11A1)	F: AGA AGC TGG GCA ACA TGG AGT CAG<br>R: TCA CAT CCC AGG CAG CTG CAT GGT	J05156	Orly et al. 1994
StAR	F: CAT CCA GCA AGG AGA GGA AG<br>R: CGT GAG TTT GGT CTT TGA GG	AY736357	Kim et al. 2002
3β-HSD	F: ACT GGC AAA TTC TCC ATA GCC<br>R: TTC CTC CCA GCT GAC AAG TGG	M38178	Orly et al. 1994
CYP17A1	F: GCC TGA CGG ACA TTC TG<br>R: TCG TGA TGC AGT GCC CAG	MM_012753	Livera et al. 2004
